# Thyroid Cancer: From Potential Drivers to Real Modulators

**DOI:** 10.3390/cancers18152474

**Published:** 2026-08-01

**Authors:** Shafiya Imtiaz Rafiqi, Juan Carlos Jaume

**Affiliations:** 1 Department of Medicine, Edward Hines Jr. VA Hospital, Hines, IL 60141, USA; shafiyaimtiaz.rafiqi@va.gov; 2 Department of Medicine, Loyola University Chicago, Chicago, IL 60660, USA

**Keywords:** thyroid cancer, tumor immune microenvironment (TIME), immunotherapy, tumor associated macrophages

## Abstract

The advent of cancer immunotherapy opened a transformative era in oncology, shifting the focus from targeting mutated genes through precision oncology to harnessing the body’s immune system via agnostic therapies. This paradigm has demonstrated that a deeper understanding of the tumor immune microenvironment (TIME) can transcend the challenges posed by cancer’s genetic diversity and evolutionary dynamics. In the case of thyroid cancer, progress in immunotherapy has been comparatively slow. Much of the current research still centers on the genetic landscape of thyroid tumors rather than on comprehensive exploration of their TIME. The TIME, composed of immune cells such as macrophages, lymphocytes, natural killer cells, and mast cells, along with a network of signaling molecules, evolves alongside tumor growth and metastasis. It both influences and is influenced by genetic alterations, metabolic conditions, and therapeutic interventions. This dynamic crosstalk defines the clinical and biological heterogeneity of thyroid cancers; from the aggressive anaplastic thyroid carcinoma (ATC) to the more indolent papillary thyroid carcinoma (PTC). Mapping the spatial and temporal changes within the TIME offers the opportunity to design therapies that counter immune evasion and enhance treatment response. As understanding of the tumor microenvironment deepens, thyroid cancer therapy is undergoing a major shift; from strategies aimed merely at genetic mutations to integrated, combinational approaches incorporating personalized immunotherapy. Building on recent findings, which detail immune cell composition and therapeutic implications in thyroid malignancies, this review expands on the molecular and cellular mechanisms shaping the microenvironment. We highlight how oncogenic signaling, stromal remodeling, and metabolic reprogramming coordinate to influence tumor immunity, and we explore emerging strategies that aim to reengineer the tumor microenvironment for improved therapeutic outcomes.

## Simple Summary

Unleashing the immune microenvironment to cure cancer regardless of its genetic origin was once an unimaginable concept that is now a groundbreaking reality. While the notion of immunotherapy is not new, the modern era has seen a dramatic shift from targeting tumors by their genetic make-up to targeting the molecular features that fuel their growth in the tumor microenvironment—an approach known as “agnostic” therapy. This paradigm shift was made possible by decades of research into the intricate relationship between cancer cells and the surrounding tumor immune microenvironment (TIME). Scientists uncovered how cancer cells exploit and manipulate the TIME to evade the immune system, leading to the development of therapies that block these escape mechanisms. We discuss here many of the genetic drivers thought to bring about thyroid cancer and immune modulators that allow or preclude its existence.

## 1. Introduction

In the late 19th century, surgeon William Coley noticed that some cancer patients experienced tumor regression after bacterial infections, though the mechanism was unknown. This was the earliest hint that the immune system could be directed against cancer. In the mid-20th century, the theory of cancer immunosurveillance proposed that the immune system acts as a natural protective mechanism against cancer. However, this idea was disregarded for many years due to a lack of evidence. The discovery of key immune cells (like cytotoxic T-cells and natural killer cells) and messenger molecules (like cytokines) in the 1960s and 1970s provided the foundational knowledge for manipulating immune responses. The crucial breakthrough came with the discovery and characterization of immune checkpoint molecules like CTLA-4 and PD-1 in the 1990s; blocking these with immune checkpoint inhibitors (ICIs) could release the brakes on the immune system and allow it to attack cancer cells. The true paradigm shift happened when researchers discovered that certain genomic features, specifically expression of immune checkpoint ligands, made a tumor vulnerable to ICIs, regardless of the cancer genetic make-up (oncogenes and/or mutated tumor suppressor genes). 

The development of cancer immunotherapy marked a new chapter in oncologic treatments which went from targeting mutated genes (precision oncology) to unleashing tumor immunity (agnostic therapy) and proved that a deeper understanding of the TIME could overcome the dynamic genetic and constantly changing diversity of cancer itself. 

When it comes to Thyroid cancer, the field has had a slow start with regards to immunotherapy. As discussed here, much attention is still focused on the genetic make-up of thyroid tumors as opposed to investigation of its TIME.

Building on recent work [1,2] that comprehensively described the immune cell populations and their changing composition across thyroid cancer subtypes, the present review focuses on the connecting oncogenic drivers to the immunosuppressive microenvironment they create and critically assesses the evidence supporting therapeutic approaches designed to overcome these changes. We argue that deeper understanding of thyroid cancer immunology depends not only on identifying the immune cells that are present, but also on explaining the molecular and cellular processes that determine their function, with particular emphasis on signaling networks, metabolic changes, and interactions between tumor, stromal, and immune cells. In addition, this review presents the Src, JAK–STAT, and Wnt pathways as interconnected contributors to immune evasion and treatment resistance, rather than as independent oncogenic pathways. Viewing these pathways in relation to one another provides new insight into the development of resistance and highlights opportunities for more rational combination therapeutic strategies. We additionally examine the emerging gut–thyroid axis, arguing that intratumoral microbiota and gut microbial metabolites are active participants in thyroid tumor metabolic reprogramming and immune suppression, with active modulation of glycolytic reprogramming, sodium iodide symporter expression, and CD8+ T-cell function within the thyroid tumor microenvironment. Finally, we systematically differentiate evidence derived from in vitro models, animal systems, and clinical datasets to identify which therapeutic hypotheses didn’t proceed beyond in vitro models and cell lines, which are preclinically supported, and which remain clinical speculation.

## 2. Literature Search Strategy

This review aims to provide an integrated overview of thyroid cancer biology encompassing the cellular architecture of the TIME, genetic drivers and signaling pathways underlying its evolution, current therapeutic strategies designed to overcome immunosuppression and the gut–thyroid axis. These domains are well covered individually in the existing literature but less often synthesized together. The literature search was conducted through PubMed/MEDLINE, Scopus, and the Cochrane Library for English-language articles, published from January 2000 to December 2025 to also capture recent conference abstracts and preprints relevant to emerging therapeutic strategies. Search terms were constructed as follows: thyroid cancer histological subtypes (papillary thyroid carcinoma, follicular thyroid carcinoma, medullary thyroid carcinoma, poorly differentiated thyroid carcinoma, anaplastic thyroid carcinoma) were combined with tumor microenvironment and immune terms (tumor immune microenvironment, TIME, tumor-associated macrophages, regulatory T cells, MDSCs, immune checkpoint, PD-L1, PD-1, TILs), and with molecular pathway and therapeutic terms (BRAFV600E, RAS, RET, TERT, MAPK, PI3K/AKT/mTOR, Wnt/β-catenin, JAK-STAT, Src, TGF-β, STING, TLR, metabolic reprogramming, Warburg effect, IDO1, gut microbiome, cancer-associated fibroblasts, tumor stroma). Clinical trial data were retrieved from ClinicalTrials.gov.

## 3. General Homeostatic Disruptions Initiate Thyroid Cancer

The thyroid gland is a very metabolically active organ and the follicular cells under control of the hypothalamic–pituitary–thyroid axis are tightly regulated in their growth and function via the thyrotropin (TSH) receptor pathway and downstream signaling cascades like MAPK and PI3K/AKT. Oncogenesis appears to initiate when these follicular cells acquire genetic mutations such as BRAF^V600E^, RAS, RET/PTC rearrangements, or PAX8-PPARG fusions; that constitutively activate signaling pathways, leading to uncontrolled cellular growth (Table 1). Environmental factors like radiation exposure and inherited conditions like multiple endocrine neoplasia type 2 (MEN2) further elevate this risk. The transformation from a normal cell to a malignant phenotype also involves epigenetic modifications (e.g., DNA methylation, TERT promoter mutations) that promote dedifferentiation and are linked to therapy resistance. As cancer advances, additional alterations, such as p53 mutations, confer a more aggressive, treatment-resistant phenotype. The camouflage and sabotage theory offers a conceptual framework for understanding how cancer cells evade immune detection and actively suppress immune function [3]. In the camouflage phase, tumor cells reduce antigen visibility, mimic host molecules, and downregulate MHC molecules. In the sabotage phase, tumors hijack immune regulatory pathways (e.g., PD-L1 expression), secrete immunosuppressive cytokines (e.g., IL-10, TGF-β), and recruit suppressive immune cells such as regulatory T cells (Tregs) and M2 tumor associated macrophages (TAMs). These processes collectively reshape the tumor microenvironment into a pro-tumoral niche. Each thyroid cancer subtype reflects a distinct constellation of molecular drivers that govern its behavior, progression, and responsiveness to treatment. The thyroid cancer TIME is shaped by a plethora of factors including genetic drivers (BRAF, RET, TERT), pathways (PD-1, TGF-β, TLR, STING, WNT, MAPK), cellular interactions (TAMs, Tregs, MDSCs), stromal/ECM components (collagen/cancer-associated fibroblasts, CAFs), metabolic reprogramming, and cytokine dynamics.

### Tumor Microenvironment Architectural Disruption and Key (Non-Immune) Cellular Components

The tumor stroma includes cellular elements such as immune cells (e.g., mast cells, macrophages, T cells) [1], endothelial cells, pericytes, and non-cellular components like the extracellular matrix (ECM), soluble factors (e.g., cytokines, chemokines, growth factors), and vascular structures. Cellular compositions and interactions at the interphase of nonmalignant and tumor tissues have significant effects on tumor microenvironment remodeling and likely influence tumor initiation, progression, metastasis, immune evasion, and response to therapy. In direct cell–cell contact and indirect mechanisms, key cellular components, such as endothelial cells, pericytes and adipocytes support and facilitate tumor cells architectural disruption of protective tumor immunity provided by the tumor ymicroenvironment.

**a.**  **Endothelial cells:** Endothelial cells, along with pericytes, form the inner lining of blood vessels and contribute to the vascular network essential for nutrient and oxygen supply in tumors. In thyroid cancer, endothelial cells are central to the angiogenic switch, particularly in aggressive subtypes like ATC and DDTC, where increased microvascular density correlates with poor prognosis. These cells are activated by hypoxia-induced factors such as HIF-1α and pro-angiogenic signals like VEGF, FGF2, and PDGF [4]. They secrete cytokines and express adhesion molecules (VCAM-1, ICAM-1) that regulate immune infiltration, particularly T cell and NK cell, often contributing to immune evasion via PD-L1 expression [5]. Endothelial cells also interact with tumor-associated macrophages and mast cells to amplify angiogenesis and, under certain conditions, may undergo endothelial-to-mesenchymal transition (EMT), enhancing ECM remodeling [6]. MALAT1-driven FGF2 secretion by TAMs further boosts endothelial activity [7]. Therapeutically, anti-angiogenic agents such as sorafenib and cabozantinib have been approved to inhibit VEGF signaling and reduce tumor vascularization [7]. Ongoing research is investigating combination therapies, pairing these agents with immune checkpoint inhibitors addressing both angiogenesis and immune suppression [8].
**b.**  **Pericytes:** Pericytes are mural cells that envelop endothelial cells and play a critical role in stabilizing and maturing blood vessels. In thyroid cancer, they are involved in maintaining vascular integrity and promoting angiogenesis. Their stem-cell-like properties allow them to differentiate into multiple cell types, contributing to tumor heterogeneity [9]. Pericytes interact with endothelial cells to regulate vessel function and modulate the tumor microenvironment through the secretion of cytokines and growth factors. In vitro co-culture studies suggest that pericytes in BRAFV600E-positive PTC release thrombospondin-1 (TSP-1) and activate TGF-β1, shielding tumor cells from tyrosine kinase inhibitors [9]. Furthermore, pericytes influence immune modulation and engage in crosstalk with CAFs and immune cells. They express platelet-derived growth factor receptor-β (PDGFR-β), making them a potential target for combinatorial therapies but warranting a subtype-specific approach, as different thyroid cancer subtypes may differ in pericyte abundance [10].
**c.**  **Adipocytes:** Cancer-associated adipocytes are emerging key contributors to cancer types that regulate energy homeostasis and secrete bioactive adipokines, including leptin, adiponectin, TNF-α, and IL-6. Within the thyroid cancer microenvironment, adipocytes—particularly cancer-associated adipocytes (CAAs)—play a significant role in tumor development and progression. They contribute to a pro-tumorigenic milieu by enhancing chronic inflammation, remodeling the ECM, and attracting myeloid cells through chemokine secretion [11]. Leptin activates the JAK2-STAT3 and MAPK pathways, promoting cancer cell proliferation and metastasis, while adiponectin—typically downregulated in obesity—exerts anti-tumor effects via AMPK activation and PI3K-AKT-mTOR inhibition [12]. The balance between these adipokines shapes disease outcomes. Obesity intensifies these effects, with epidemiological data showing increased thyroid cancer risk and aggressiveness in obese individuals [13]. However, these associations are confounded by BMI measurement errors and unadjusted thyroid-stimulating hormone (TSH) levels, which may independently affect cancer development. Preclinical studies using mouse xenografts demonstrate that AdipoRon, an adipocyte-targeting agent, inhibits PTC growth via the AdipoR2-ULK/p-ULK1 axis [12]. These models, however, do not fully mimic human microenvironments, limiting their applicability. Clinical translation of adipocyte-targeted therapies will require more robust human-relevant data and is still in its early stages.

## 4. Molecular Drivers and Their Tumor Microenvironment Effects

Genetic and molecular drivers: Oncogenic alterations in thyroid cells such as BRAF^V600E,^ RAS, and TERT promoter mutations, TP53 mutations, PIK3CA and RET/PTC rearrangements initiate tumor formation and modulate the TIME by altering cytokine production, antigen presentation, and immune checkpoint expression.

**a.**  **BRAF^V600E^:** BRAF gene belongs to the RAF (Rapidly Accelerated Fibrosarcoma) family, located on chromosome 7 and is classified as protooncogene. The B-Raf protein is a crucial component of a vital cellular signaling pathway known as the RAS-RAF-MEK-ERK (or MAPK, mitogen-activated protein kinase) pathway. A point mutation in the BRAF gene called BRAF^V600E^, constitutively activates the MAPK pathway, driving tumor proliferation, increased cancer cell survival, enhanced invasion and metastatic potential. BRAF mutations are found in 36–69% of PTC and 20–45% of ATC and testing for BRAF^V600E^ mutation in FNA samples is routinely performed to aid in diagnosis when cytological findings are indeterminate [14]. Of note, the different diagnostic threshold of PTC nuclear features resulted in a high (50–90%) incidence of BRAF^V600E^ mutation in PTCs in most Asian countries, whereas it was lower (35–50%) in most Western patient cohorts [15]. However, these may stem from variations in cytological interpretation rather than inherent biological differences. Human studies, including IHC and TCGA data, support the association between BRAF^V600E^ and PD-L1 expression in patient tissues, while in vitro and animal studies provide mechanistic insights into MAPK-dependent transcription, immune cell recruitment, and TAM polarization. TCGA and IHA analysis associate BRAF^V600E^ with upregulated PD-L1 expression in patient tumor cells and tumor-associated macrophages (TAMs) while in vitro studies implicate MAPK-dependent transcription, inhibiting T-cell cytotoxicity. This augments immunosuppressive cytokine milieu in the TIME compounded by regulatory T-cell (Treg) and myeloid-derived suppressor cell (MDSC) recruitment, and polarizing TAMs toward an M2-like (pro-tumorous) phenotype. It modulates the stroma by enhancing thrombospondin-1 (TSP-1) expression in pericytes present in the tumor microenvironment, activating TGF-β and increasing extracellular matrix (ECM) stiffness, which further limits immune cell infiltration. BRAF/MEK inhibitors have successfully been used to treat thyroid cancer. BRAF inhibitors initially show efficacy in BRAF-mutant thyroid cancers, but resistance often develops through MAPK reactivation (e.g., KRAS/NRAS mutations, BRAF splice variants), PI3K/AKT/mTOR pathway activation (via IGF-1R upregulation or PTEN loss), and tumor microenvironment factors like hypoxia-induced HIF-1α and immune evasion. These multifactorial resistance mechanisms necessitate combinatorial strategies (e.g., BRAFi + MEKi ± PI3Ki/immunotherapy). Dabrafenib (BRAF inhibitor) and trametinib (MEK inhibitor) are FDA-approved for BRAF-mutated ATC. Results from an ongoing Phase II single arm multicenter trial reveal that a pre-surgical combination therapy including pembrolizumab plus dabrafenib and trametinib (DTP) significantly improved survival in patients with rare BRAF^V600E^-mutated ATC, and adding ICI to the BRAF/MEK inhibitors enabled tumor resection in the majority of patients (74%), compared to just 5% historically [16]. However, the historical resection rates in ATC reflect a patient population that was not selected by BRAF mutation status, whereas the trial enrolled only BRAF-mutant patients, who may represent a biologically distinct, more treatment-responsive subset irrespective of the specific regimen. The absence of a control arm means we cannot attribute the resection rate to the pembrolizumab addition specifically, dabrafenib/trametinib alone has demonstrated meaningful responses in BRAF-mutant ATC. While promising, the trial’s single-arm design, small sample size, and absence of long-term survival data underscore the need for larger, randomized controlled studies to validate these findings. In earlier clinical trials while responses in PTC are relatively durable (median progression-free survival >15 months), outcomes in ATC remain limited, with a median overall survival of approximately 14 months despite a 56% objective response rate. Although phase III placebo-controlled data from trial NCT04554680 confirm therapeutic benefit, they exclude patients with aggressive histologic subtypes. Therefore, what the trial does establish convincingly is that neoadjuvant triple therapy is feasible and safe in this population; what it cannot establish is the marginal contribution of pembrolizumab to surgical conversion, the durability of response after resection, or generalizability beyond BRAF-mutant ATC. Comparative studies with mature survival endpoints will be required to determine whether triple therapy offers a meaningful advantage over targeted therapy alone.
**b.**  **RAS:** Like BRAF^V600E^, RAS mutations in thyroid cancer are typically point mutations occurring at specific “hotspot” codons (most commonly codons 12, 13, or 61) [17]. Their prevalence varies across thyroid cancer subtypes and a significant number of follicular adenoma (20–40%), noninvasive follicular thyroid neoplasm with papillary-like nuclear features (NIFTP) (30–60%), uncertain malignant potential (UMP) (14–20%), follicular variant PTC (15–43%), FTC (30–50%) and poorly differentiated thyroid carcinoma (18–50%) cases have RAS mutations [15]. Overall, RAS mutations predominate in FTC whereas mutant BRAF is much more frequent in PTC. Notably, distant metastasis is a typical feature of FTC but is much less frequent in patients with PTC. Thus, mutation identity (RAS vs. BRAF) is more important for tumor invasiveness and spreading and, importantly, the specific level of activity these mutations induce within the MAPK signaling pathway [18]. Mechanistically, RAS mutations differ from BRAF^V600E^ and TERTp mutations by decreasing sodium iodide symporter and TSHR expression and RAI uptake through AKT activation [19]. RAS mutations constitutively activate the MAPK pathway (via ERK) and PI3K/AKT pathway (via mTOR), driving tumor proliferation, survival, and invasion. These pathways upregulate immunosuppressive molecules like VEGF, TGF-β, CCL2, and CXCL12, which recruit pro-tumorous immune cells and inhibit effector immune responses. RAS mutations are associated with a less immunogenic TIME than BRAF^V600E^ due to lower PD-L1 induction, contributing to “cold” TIMEs, explaining their relative resistance to single-agent ICIs [20]. While it seems biologically coherent, these findings rely primarily on transcriptomic and IHC data rather than ICI trials stratified by RAS status. Available clinical datasets are scarce and lack systematic RAS-stratified reporting. Critically, “cold” and “hot” describe a continuous spectrum: RAS-mutant tumors with intermediate PD-L1 and modest CD8+ infiltration may respond better to combination ICI plus antigen-boosting strategies than to anti-PD-1. Moreover, RAS mutations exert their oncogenic effects additionally through activating other signaling pathways, which makes drug development targeting of RAS more challenging and provides a logical basis for explaining differing biological effects and outcomes of RAS vs. BRAF driver mutations in the thyroid [14].
**c.**  **RET mutations/fusions:** A RET mutation is an alteration in the RET (Rearranged During Transfection) proto-oncogene, which encodes a receptor tyrosine kinase protein critical for cell growth and nerve cell development. RET mutations are found in PTC and MTC, however, the activation mechanisms are different in the two types. Chromosomal rearrangements are found in PTC, and somatic mutations lead to MTC [21]. The general mechanisms of aberrant RET activation include in-frame RET fusions, targeted mutation, and aberrant overexpression that trigger the PI3K-AKT-mTOR and RAS-RAF-MEK-ERK pathways [22]. RET/PTC chimeric protein form dimers that are required for oncogenic activation, which activate the RAS/MAPK/ERK pathway to promote proliferation and migration of thyroid cancer cells. RET fusion has been reported more commonly in pediatrics and dose-dependently with irradiation. Radiation exposure is not associated with RET fusion in MTC compared to PTC. The therapeutic landscape for advanced MTC has undergone a significant evolution over the past decade, transitioning from a state of multikinase inhibitor based treatment to a highly stratified, biomarker-driven paradigm centered on selective RET inhibition, with the objective response rates (ORR) being ~80% with selective RET inhibitors [21]. Although, acquired resistance mechanisms via alternative pathways activation (e.g., Src) tempts for future combinatorial strategies, yet evidence supporting Src-mediated resistance derives predominantly from pre-clinical cell line and patient-derived xenograft models with no clinical validation, casting probable doubts on its translational maturity. In contrast, on-target RET solvent-front mutations (particularly G810 substitutions) represent the best-established mechanism of acquired resistance.
**d.**  **TERT:** TERT (telomerase reverse transcriptase) promoter mutations reactivate the expression of telomerase, a recognized oncogene normally quiescent in differentiated adult human cells. While TERT promoter mutations are present in less than 10% of indolent PTCs, their prevalence markedly increases in high-grade PTCs and poorly differentiated thyroid cancers (PDTCs), reaching over 70% in highly aggressive ATCs [23]. It needs to be emphasized that tumors with BRAF^V600E^ and TERTp mutations exhibit higher signaling activation in the MAPK signaling pathway than those with RTK or RAS mutation alone, promoting a more invasive phenotype in PTC. One possible reason could be that TERT mutations suppress the intrinsic immune-activating properties of BRAF and unleash the oncogenic potential of the constitutively active BRAF-MAPK signaling pathway. Nevertheless, the synergistic effect of BRAF^V600E^ and TERTp represents a critical prognostic marker, aiding in the identification of patients with higher mortality risk. P53 activation indicates the last step in tumor progression. When TP53 is mutated, it triggers uncontrolled cell replication and tumor development. Double mutated PTC (BRAF^mut^pTERT^mut^) show significantly repressed transcripts for genes dictating the immune landscape of PTC like PTPRC/CD45, and CD45 effector kinase, LCK, cytotoxicity markers GZMB, NK cell markers, KLRB1, KLRC1 and KLRD, CALCA/calcitonin and T-cell chemokine, CCL21 [24].
**e.**  **Tumor mutation burden (TMB):** TMB is a quantitative measurement of the total number of somatic non-synonymous mutations in each coding area of the tumor genome. Thyroid cancer has the third lowest TMB (TMB between 0.1 and 1 mut/Mb) [25]. Since neo-antigens are produced because of mutations, the greater the number of mutations (i.e., the higher the TMB), the greater the chance that some of the neo-antigens presented by MHC proteins will be immunogenic and hence enable T-cell recognition and cancer cell eradication. However, for mutations to be translated to neoantigens and elicit an immune response, the altered neo antigens should be presented by the MHC and recognized by TCRs. In thyroid cancer, high TMB in ATC (~5–10 mut/Mb) generates neoantigens, but many are non-immunogenic due to MHC loss, poor antigen presentation and counteracted by PD-L1, TGF-β, IL-10, and the presence of Tregs, M2 TAMs, and MDSCs, creating “immune-deserted” TIMEs.

## 5. Metabolic Reprogramming

As thyroid tumors exceed 1–2 mm in size, they require new vasculature to meet increased metabolic demands. Metabolic reprogramming, a hallmark of thyroid cancer, involves a shift to aerobic glycolysis (Warburg effect), enhanced glutaminolysis, lipid synthesis, and amino acid metabolism [26]. Hypoxia-inducible factor-1α (HIF-1α), mitochondrial ROS, and hormones like TSH and TH are key regulators of these metabolic shifts [27]. HIF-1α directly enhances glycolysis by upregulating GLUT1, and Yes-associated protein (YAP) overexpression further augments glycolytic gene expression, histone acetylation (e.g., H3K27ac), and EMT. This cascade creates an acidic microenvironment conducive to metastasis. Acidification impairs T and NK cell function and promotes regulatory T cell differentiation, suppressing antitumor immunity [28]. Cancer cells compete with immune cells for glucose and glutamine, shifting TAMs to pro-tumoral M2 phenotypes. Endothelial cells respond to HIF-1α by upregulating GLUT1 and PFKFB3, promoting angiogenesis. CAFs switch to glycolysis and secrete lactate and glutamine (reverse Warburg effect), supporting tumor metabolism [29]. In parallel, tumors deplete nutrients such as glucose and glutamine, starving immune cells, and activate tryptophan catabolism through overexpression of indoleamine 2,3-dioxygenase (IDO1), resulting in accumulation of kynurenine and tryptophan depletion. This metabolic axis suppresses CD8+ and NK cell function while promoting regulatory T cell (Treg) expansion via aryl hydrocarbon receptor (AhR) signaling. Immune cells, including TAMs and neutrophils, reprogram to favor glycolysis and fatty acid synthesis. These metabolic adaptations differ across thyroid cancer subtypes: PTC and FTC show heightened glycolysis and glutaminolysis, with nutrient-depleted TIMEs, impairing immune responses. ATC is profoundly hypoxic with elevated GLUT1 and lactate production, driving angiogenesis and immune evasion. MTC shows alterations in amino acid metabolism, contributing to immune modulation. Prognostically, high expression of glycolytic markers (e.g., GLUT1, LDHA), lipid enzymes (e.g., SCD1), and glutamine-related molecules (e.g., GLS1) correlates with worse outcomes. Therapeutic interventions targeting these pathways, including glycolysis inhibitors (2-DG), glutaminase inhibitors (CB-839), and IDO inhibitors, along with combination therapies, hold promise for overcoming resistance and reshaping the metabolic landscape of the tumor microenvironment [30]. IDO1 inhibitors like epacadostat, in combination with PD-L1 blockade, (e.g., ECHO-202 trial) reported objective response rates up to 55% in immunotherapy-naïve patients [31]. However, extrapolation to thyroid cancer presented with serious caveats. First, epacadostat subsequently failed in a large, randomized phase III trial (KEYNOTE-252) in melanoma; highlighting complexity of immunometabolic regulation, and raising fundamental questions about whether IDO1 inhibition meaningfully contributes to clinical ICI responses or whether ECHO-202’s ORR reflected pembrolizumab’s activity alone. In thyroid cancer, overexpression of IDO1 is associated with increased Treg infiltration and immune escape, especially in BRAF-mutant PTC and ATC, suggesting that similar checkpoint metabolism interactions occur, but the findings are based on single-institution IHC studies with limited sample sizes and no consensus on scoring methodology, making prevalence estimates unreliable. Therefore, extrapolating from these proof-of-concept studies, the Metabolic-Immune Circuit Breakers such as GLS1/PD-1, IDO1/PD-L1 etc. offer a plausible hypothesis for a thyroid specific preclinical validation.

## 6. Hormonal Influences on Thyroid Cancer Tumor Microenvironment

Hormonal differences, particularly estrogen and androgen levels, are central to understanding tumor microenvironment variations. Sex hormones function as key modifiers of tumor evolution rather than primary mutagens. Estrogen significantly impacts the female thyroid cancer microenvironment, promoting tumor growth and vascularization. Females having higher concentration of estrogens than males exhibit increased expression of estrogen receptors (ERα and ERβ) in thyroid cancer cells, particularly in RET/PTC. This promotional drive is executed via an increased ERα/ERβ ratio, downregulating the protective PPAR γ ligand pathway, and GPER-mediated transactivation of EGFR to engage the MAPK and PI3K/Akt cascades. Somatic driver mutations, specifically the BRAF^V600E^ alteration, actively reprogram the thyroid cell’s transcriptional response to estrogen, converting E2 from a homeostatic, differentiation-promoting hormone into a potent driver of migration, invasion, and anoikis-resistant metastatic growth. Studies show that estrogen upregulates ERα, enhancing cell proliferation, migration, and invasion via pathways like ERK1/2 [32]. Additionally, estrogen increases vascular endothelial growth factor (VEGF) secretion, modulating the vascular environment to support tumor growth [33]. Female thyrocytes also exhibit higher reactive oxygen species (ROS) production and lower antioxidant capacity, potentially increasing DNA damage and creating a more oxidative microenvironment. However, in males, androgens, such as testosterone, can promote PTC cell growth, invasion, and epithelial–mesenchymal transition (EMT) via the P38/JNK pathway. Conversely, in studies based on androgen-responsive thyroid cancer cell line, androgen receptor (AR) activation may reduce tumor aggressiveness by decreasing PD-L1 expression via inhibition of the nuclear factor kB (NF-kB) signaling and inducing senescence [34]. This hormonal dimorphism necessitates sex-specific considerations in studying tumor microenvironment biology and therapy response. Sex-specific therapy trials in thyroid cancer are essentially nonexistent, meaning that the clinical translation pathway for any of these hormonal insights remains undefined. Most of the findings are based on in vitro studies using supraphysiological hormone concentrations in cell lines that may not reflect the receptor expression profiles of primary tumors. Clinical epidemiological data consistently show female predominance in thyroid cancer incidence but not in disease-specific mortality, which complicates the inference that estrogen is a meaningful driver of aggressive behavior versus overdiagnosis of indolent disease.

## 7. Pathway Networks and Crosstalk

**Figure 1:**
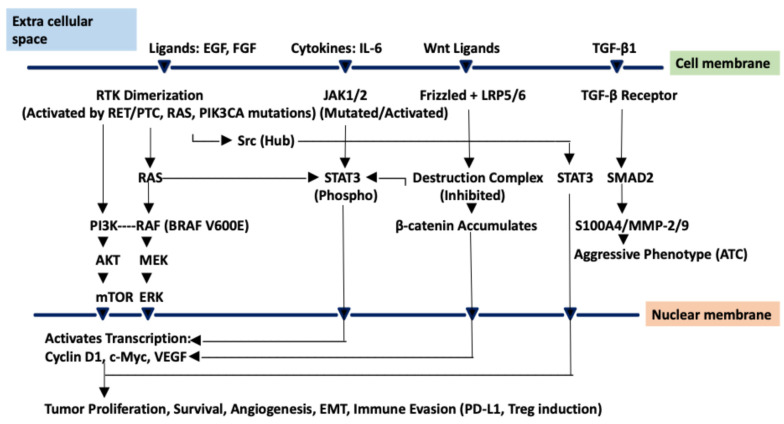
Cellular pathways.

Several core-signaling pathways orchestrate communication between thyroid cancer cells and the tumor microenvironment, modulating proliferation, immune evasion, angiogenesis, and stromal remodeling (Figure 1). MAPK activation is crucial for PTC initiation, while PI3K/AKT activation is thought to be critical in FTC initiation, establishing a fundamental difference in pathway dependence between subtypes. This pathway-centric classification has revolutionized thyroid cancer understanding and treatment, moving from purely histological to molecular-based approaches (Table 1).

**a.**  **MAPK (Mitogen-Activated Protein Kinase) pathway**: The MAPK pathway is a central signaling cascade in thyroid cancer, especially in PTC. It is composed of key kinases such as RAS, RAF, MEK, and ERK and is typically activated by the stimulation of membrane-bound tyrosine kinase receptors through ligands like fibroblast growth factor (FGF) and epidermal growth factor (EGF) [35]. Ligand binding leads to receptor dimerization and autophosphorylation, initiating intracellular signaling. Genetic alterations such as BRAF^V600E^ mutations, RAS mutations, and RET/PTC rearrangements result in constitutive pathway activation, promoting tumor proliferation, survival, angiogenesis, and metastasis. MAPK signaling influences the TIME by upregulating VEGF and modulating cytokine and chemokine secretion, which supports angiogenesis, immune evasion, and epithelial-mesenchymal transition (EMT). It can also promote the development of immunosuppressive regulatory Tregs. Additionally, IL-6 from tumor-associated M2 macrophages can activate both MAPK and JAK/STAT3 signaling, contributing to PD-L1 expression and tumor immune escape [36]. The pathway’s activation has been implicated in aggressive phenotypes across PTC, FTC, PDTC, and ATC. Therapeutic strategies targeting this pathway include RAF and MEK inhibitors such as vemurafenib and sorafenib, which remain under clinical evaluation for advanced thyroid cancers. Inhibition (BRAF/MEKi) not only suppresses tumor growth but also reverses TIME immunosuppression, validating it as a keystone pathway for combinatorial immunotherapy.
**b.**  **PI3K/AKT/mTOR pathway:** The PI3K/AKT signaling pathway plays a critical role in thyroid cancer progression by transmitting signals from receptor tyrosine kinases to downstream effectors involved in cell proliferation, survival, and metabolism. In PTC, dysregulation of this pathway occurs via various mechanisms including circ_PSD3 sponging miR-637 to enhance HEMGN and PI3K/AKT activity [37], and TEKT4-mediated microtubule stabilization promoting cell motility and invasion [38]. Also, microRNAs like miR-30b-5p suppress PTC progression by targeting GALNT7, thereby inhibiting the EGFR/PI3K/AKT axis. Mutations in PIK3CA and loss of PTEN are prevalent in ATC and PDTC, contributing to recurrence and metastasis. The pathway also shapes the tumor microenvironment by upregulating VEGF, supporting angiogenesis, modulating immune responses, and driving metabolic reprogramming. Therapeutic efforts targeting this axis include PI3K inhibitors (e.g., GDC-0941) and Akt inhibitors (e.g., MK-2206), especially in combination regimens for advanced thyroid cancers [39]. Additionally, the crosstalk with MAPK underscores the need for vertical pathway inhibition in refractory disease.
**c.**  **Wnt/β-catenin signaling pathway**: The Wnt signaling pathway plays a complex and multifaceted role in thyroid cancer, influencing key processes such as cell proliferation, differentiation, and metastasis. It primarily functions through two main branches: the canonical (Wnt/β-catenin) pathway and the non-canonical pathways. The canonical pathway is activated by Wnt ligand binding to Frizzled and LRP5/6 receptors, inhibiting a “destruction complex” and leading to β-catenin accumulation and nuclear translocation, where it activates gene transcription promoting cell proliferation. Non-canonical pathways, such as Wnt/PCP and Wnt/Ca2+, are β-catenin-independent and regulate cellular polarization, migration, and cytoskeletal organization [40]. While the canonical Wnt/β-catenin pathway is often associated with oncogenic activity, promoting cell survival and growth, certain Wnt ligands, such as Wnt5a, can display a paradoxical tumor-suppressive function in specific thyroid cancer contexts [41]. In the canonical Wnt/β-catenin pathway, mutations like those in CTNNB1 lead to β-catenin accumulation, driving tumor growth through oncogenes like Cyclin D1 and c-Myc; however, in ATC, canonical Wnt activation is instead driven predominantly by ligand overexpression. Furthermore, non-canonical pathways, such as Wnt/Ca2+ involving Wnt5a, exhibit context-dependent effects, acting as a tumor suppressor in FTC by inhibiting proliferation via CaMKII-mediated β-catenin degradation, while promoting metastasis in PTC through interactions with molecules like lncRNA FAM230B [42]. The pathway’s crosstalk with Notch, NF-κB, and STAT3, along with modulation by extracellular matrix components like Tenascin-C (TNC) and epigenetic changes such as DNA methylation, further shapes the tumor microenvironment, contributing to angiogenesis, immune evasion, and invasion. Because ligand-dependent Wnt activation remains susceptible to upstream inhibition, Porcupine inhibitors or receptor-directed strategies provide a biologically plausible therapeutic approach for ATC compared to mutation-targeted strategies. Targeting Wnt signaling offers therapeutic potential in multiple solid tumors, with strategies like Porcupine inhibitors (e.g., WNT974), monoclonal antibodies (e.g., Vantictumab), and epigenetic modulators (e.g., HDAC inhibitors) under investigation [43], but challenges include systemic toxicities due to Wnt’s role in normal tissues and tumor heterogeneity, necessitating subtype-specific approaches. The lack of thyroid cancer-specific Wnt-targeted clinical trials highlights a translational gap, underscoring, perhaps, the need for precise molecular diagnostics and personalized therapies to address Wnt pathway aberrations effectively (ligand driven vs. mutation driven).
**d.**  **JAK-STAT pathway:** The JAK-STAT (Janus Kinase–Signal Transducer and Activator of Transcription) pathway is a highly conserved and critical signaling cascade that transmits extracellular cues primarily cytokines and growth factors to the nucleus to regulate gene expression. It involves ligand binding to cell-surface receptors, activation of associated JAK kinases, and subsequent phosphorylation of STAT proteins, which dimerize and translocate to the nucleus to modulate transcription. Its dysregulation drives oncogenesis through enhanced cell proliferation (via STAT3/STAT5 activation of genes like cyclin D1 and c-Myc), inhibition of apoptosis, angiogenesis (via VEGF), metastasis, immune evasion, and support of cancer stem cells [44,45]. The pathway is activated by mechanisms like JAK1 mutations (ATC, PDTC), Nav1.6-mediated JAK2-STAT3 activation in FTC [46], and BRAF inhibitor (BRAFi) resistance in BRAFV600E-mutant cancers, where JAK-STAT activation contributes to cancer cell survival [47,48,49], with high pathway activity linked to shorter recurrence rates and worse outcomes in PDTC and ATC. Intriguing recent studies have suggested that, in thyroid cancer, particularly concerning STAT3, the JAK/STAT3 pathway may function in the context of tumor suppression rather than promoting tumorigenesis [48,50]. Therefore, it appears that STAT3 exhibits tumor-suppressive effects in early PTC by inducing senescence via p21 upregulation [50] but becomes oncogenic in ATC through IL-6-mediated persistent activation that promotes PD-L1 expression and Treg expansion [47]. This transition correlates with P53 mutation status. Critically, p53 mutation status, which appears to gate this transition, is rarely reported as a stratifying variable in thyroid cancer studies examining JAK-STAT pathway activity. Until it is, pooled analyses of STAT3 pathway studies will continue to yield seemingly paradoxical results. Hence, a universal approach to STAT3 inhibition, which might be effective in other cancer types, could be ineffective or even detrimental and necessitate a better understanding of thyroid cancer subtypes and genetic backgrounds. Therapeutic strategies targeting JAK inhibitors (e.g., Ruxolitinib, Tofacitinib), STAT3 inhibitors (e.g., TTI-101), and combination therapies with BRAFi resistant cancers [47], are being investigated for their potential in treating advanced thyroid cancers; however, leveraging the pathway’s interactions with the TIME and other oncogenic pathways like MAPK and PI3K/AKT to guide personalized treatments would be beneficial.
**e.**  **Src signaling pathway**: The Src signaling pathway, driven by Src family kinases (SFKs), plays a pivotal role in thyroid cancer progression by regulating proliferation, invasion, angiogenesis, and immune responses within the tumor microenvironment [51,52]. Src is structurally positioned as a resistance hub, which is why it shows up as a compensatory mechanism in BRAFi-resistant PTC/ATC, in RET-Y981 signaling in MTC, and in FAK-driven invasion across subtypes. Although activating mutations in the SRC gene are rare, Src is frequently overexpressed or hyperactivated in thyroid cancers, particularly in aggressive subtypes such as ATC and MTC [53,54]. This aberrant activation often arises from upstream signals, such as RET phosphorylation at tyrosine 981 (RET-Y981), which directly activates Src and its downstream effectors, including the PI3K/Akt, MAPK, FAK, and STAT3 pathways [55,56,57]. Src interacts extensively with MAPK, PI3K/Akt, RET, and JAK-STAT pathways, driving tumor progression and resistance to therapies like BRAF inhibitors (BRAFi) in BRAF^V600E^-mutant PTC/ATC. In MTC, Src is activated by RET-Y981 phosphorylation [55,57] and, in FTC, it enhances JAK2-STAT3 signaling. SRC-3 (NCOA3), a transcriptional coactivator amplified in ATC, promotes PI3K/Akt signaling and contributes to tumor proliferation and resistance [54]. Within the tumor microenvironment, Src reshapes the immune landscape, influencing cell motility through the phosphorylation of focal adhesion kinase (FAK), and contributes to angiogenesis and immune evasion by activating STAT3 [58]. The available evidence suggests that Src functions as a convergence point that multiple driver pathways route through to execute invasion and resistance. While Src activation promotes invasion via FAK phosphorylation in tumor cores [58], its association with improved survival in THCA cohorts [59] reflects peritumoral M1 macrophage polarization that again validates spatial heterogeneity in thyroid cancers and implications in designing therapeutic approaches. Therapeutic strategies like Src inhibitors AZD0530 (Saracatinib), Vandetanib, TPX-0046, PP2, and Src-3 Inhibitor-2 (SI-2) show promise in treating thyroid cancer by targeting Src’s oncogenic activity, with varying effectiveness across subtypes like PTC, ATC, and MTC; Vandetanib’s FDA approval for MTC being a notable milestone [54,56,58,60]. Preclinical studies, such as those with AZD0530 and PP2, provide evidence for Src’s role in PTC, ATC, and MTC, while TPX-0046’s ongoing trials reflect the evolving landscape of dual-targeting strategies. These inhibitors offer hope for improving outcomes in advanced thyroid cancers, particularly by overcoming resistance to single-agent therapies like BRAF inhibitors, where Src activation serves as a compensatory mechanism. Combination strategies, such as Src inhibitors with MAPK or PI3K/Akt inhibitors, are promising, as evidenced by preclinical data.
**f.**  **TGF-β signaling pathway:** The Transforming Growth Factor-beta (TGF-β) pathway plays a multifaceted and context-dependent role in thyroid cancer, functioning as a tumor suppressor during early tumorigenesis and shifting to a tumor-promoting role in advanced disease. In healthy tissues, it inhibits proliferation via G1 phase arrest, inducing CDK inhibitors (p15INK4b, p21CIP1, p27Kip1) and reducing c-Myc, and induces apoptosis, maintaining tissue homeostasis. As cancer progresses, cells develop resistance, often through mutations in sensing receptors or SMADs, “hijacking” TGF-beta to promote EMT (loss of E-cadherin, gain of mesenchymal markers like vimentin, N-cadherin), angiogenesis (via VEGF secretion), and immunosuppression (repressing T cells, NK cells, inducing Tregs, linked to poor prognosis) [61,62]. In differentiated thyroid cancers (DTCs), PTC and FTC subtypes, this transition is enhanced by factors such as METTL7B, which correlates with poor clinical outcomes [63], and the BRAF^V600E^ mutation, which increases TGF-β secretion and represses Sodium/Iodide Symporter (NIS) expression, contributing to radioiodine resistance [64]. In ATC, TGF-β1 accelerates tumor aggressiveness through canonical (e.g., SMAD2-S100A4-MMP-2/9) signaling cascade [65]. It also remodels the tumor immune microenvironment by inducing M2-like macrophage polarization and upregulating EMT drivers such as SNAIL and SLUG, thereby enhancing metastatic potential [66,67]. The duality of TGF beta presents a therapeutic dilemma, as systemic inhibition risks disrupting tumor-suppressive functions, potentially causing inflammation or autoimmunity.

**Figure 2:**
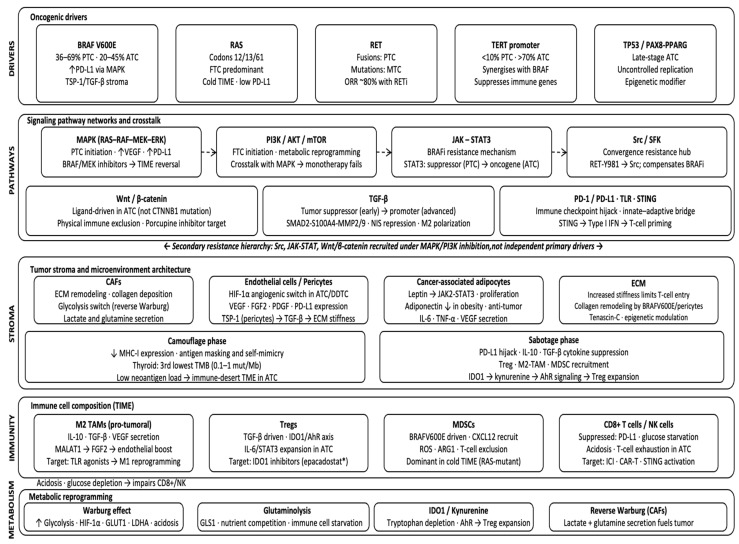
Systemic integration. The vertical downward/upward arrows within boxes indicate downregulation/upregulation. The side-ward arrows within boxes indicate directional influence. * Epacadostat failed KEYNOTE-252 phase III (melanoma). Therefore, thyroid specific IDO1/ICI trial is required before clinical translation.

Collectively, these findings underscore how thyroid tumorigenesis is orchestrated by complex interactions between its genetic drivers, various signaling pathways, epigenetic alterations, and the surrounding tumor environment (Figure 2). These pathways exhibit significant crosstalk that supports tumor growth, invasion, and metastasis while evading immune surveillance. For example, MAPK and PI3K/Akt pathways often intersect, with their activation being mutually reinforced and compensated for one another when targeted, undermining the inefficacy of monotherapies. Feedback mechanisms, such as BRAF-induced silencing of TSHR and PTEN loss, further stabilize oncogenic signaling, while combined mutations like BRAFV600E and TERT amplify MAPK activity and suppress immune-related genes, driving more aggressive phenotypes. HIF-1α and VEGF signaling interact to promote angiogenesis, while Notch and Wnt/β-catenin pathways regulate cell differentiation and EMT. NF-κB and JAK/STAT pathways contribute to a pro-inflammatory, immunosuppressive TIME, while TGF-β and estrogen signaling enhance fibroblast activation and immune suppression. This pattern is the strongest mechanistic argument in this review for combinatorial rather than sequential therapy: it implies that Src, STAT3, or Wnt inhibitors are likely to show their greatest benefit not as monotherapies but specifically when paired with the MAPK/PI3K-AKT-directed agents whose use creates the selective pressure these pathways are recruited to resist.

## 8. The Gut–Thyroid Axis: Microbiome as a Tumor Microenvironment Modulator

The discovery of intratumor microbiota/products in various cancers, including TC, has overturned the long-held belief that tumors are sterile environments. Microbial communities within tumors and the broader host microbiome, particularly the gut microbiota, are now recognized as key modulators of immune function, genetic stability, and nutrient availability. These microbial communities are not randomly distributed but are precisely structured into microniches, indicating their active involvement with immune and epithelial cell functions that influence cancer progression. The evidence leans toward metabolic regulation, including increased glycolysis, altered lipid metabolism, and lactate production, playing a pivotal role in supporting cancer cell proliferation and suppressing anti-tumor immunity [68]. Firstly, certain microbiota residing within tumors can produce genotoxins that directly damage host DNA, potentially leading to mutations that drive cancer development. Secondly, these microbial communities can significantly modulate immune responses within the TIME, either promoting or inhibiting anti-tumor immunity. Thirdly, intratumor microbiota can influence cancer progression by participating in the metabolic reprogramming of the TIME, altering nutrient availability and metabolite profiles that impact both tumor and immune cells [69]. Bacteria like Lactobacillus can promote selenium uptake, which in turn increases the transcription of the sodium/iodine symporter (NIS), a key protein for iodine uptake into thyroid cells. This suggests that Gut microbiota homeostasis may directly influence NIS function, thereby impacting overall thyroid function. Conversely, lipopolysaccharides (LPS), components of the membranes of Gram-negative bacteria, have been shown to alter NIS expression, further disrupting thyroid function [70]. Gut microbiota dysbiosis can reduce anti-tumor immunity by overstimulating CD8+ T cells, promoting chronic inflammation, and leading to early T-cell failure [71]. Bifidobacterium has been shown in animal studies to enhance the effects of anti-tumor drugs against melanoma and improve the efficacy of PD-L1 inhibitors by increasing CD8+ T-cell activity in the TIME [72]. Microbiota-derived metabolites also play a crucial role in this complex interplay. Specifically, butyrate, an SCFA, can influence iodine absorption by affecting NIS activity and can disrupt histone deacetylase (HDAC), leading to increased NIS expression [68] and sensitizing cancer cells to ferroptosis [73]. Other metabolites, such as indole-3-lactic acid (ILA) derived from Bifidobacterium breve, have been shown to ameliorate tumorigenesis by directing macrophage differentiation [74]. In contrast, certain Clostridium species metabolize primary bile acids into secondary bile acids that activate pro-oncogenic pathways, including ERK and AP-1, thereby fostering tumor progression [73]. Microbiota also orchestrate immune–metabolic crosstalk, wherein the tumor microbiome may produce metabolites that support cancer cell metabolism, such as lactate, further enhancing the immunosuppressive TIME. This bidirectional relationship underscores the complexity of the tumor microenvironment, where tumor-driven metabolic alterations reshape the composition and function of the gut microbiome. In turn, microbially derived metabolites can circulate back to the tumor microenvironment, where they actively influence tumor metabolic reprogramming and immune modulation. However, the ‘gut–thyroid axis’ hypothesis faces methodological challenges: 16S rRNA sequencing depth (median 10,000 reads/sample) detects only dominant taxa, while metabolomic studies show <5% concordance between fecal and intratumoral metabolites [68]. Recent studies collectively strengthen the association between gut dysbiosis and thyroid cancer but these studies remain observational/correlative [75,76,77]. They cannot determine if dysbiosis initiates TC, results from the tumor/metabolic changes, or is an epiphenomenon of shared confounders (diet, environment, inflammation). Nevertheless, the gut microbiota presents compelling opportunities to improve thyroid cancer therapy efficacy, with modulation through probiotics, prebiotics, and FMT explored to restore microbial diversity, reduce dysbiosis, and mitigate complications associated with thyroid hormone withdrawal in DTC patients, an important step in radioactive iodine therapy [68,78].

## 9. Therapeutic Implications and Future Directions

The intricate interplay within the thyroid cancer immune microenvironment (TIME) presents both a formidable challenge and a significant opportunity for “agnostic” therapeutic innovation. Pathways such as TLR signaling, PD-1/PD-L1, TGF-β, STING, and WNT/β-catenin are identified as prime candidates for emerging thyroid cancer therapies due to their pivotal roles in cancer immune editing and evasion. TLR agonists can effectively reprogram immunosuppressive M2 (pro-tumorous) macrophages into anti-tumor M1 macrophages, a crucial step in “heating up” immunologically “cold” tumor microenvironments, particularly those found in ATC. Reprogramming tumor-associated macrophages (TAMs) represents a promising approach, with TLR5 agonists like flagellin derivatives (e.g., entolimod) showing synergy with ICIs by enhancing CD8+ T cell priming and reversing immunosuppression. A caveat in the translation of this approach is synergy with ICIs has been tested only in immunocompetent mice; ignoring lymphocyte exhaustion in human advanced thyroid cancers. Similarly, monophosphoryl lipid A (MPLA), a safer TLR4 agonist, leverages macrophage plasticity without the systemic toxicity of LPS.

Strategies targeting the epithelial-to-mesenchymal transition (EMT) and its reversal (MET) via inhibition of key regulators like SNAIL, TWIST, STAT3, and TGF-β have shown promise in suppressing metastasis and improving responsiveness to chemotherapy and immunotherapy (NCT02423343, NCT01830621, [79]). While these approaches are advancing in other cancers, their application to thyroid cancer remains an untapped opportunity for future translational research. This translation gap may be likely due to the inherent complexity of EMT/MET dynamics and the prevailing focus on more established targets such as BRAF and RET in thyroid cancer. For example, while Snail-driven EMT link to the BRAF^V600E^ mutation and miR-222-3p has been demonstrated in papillary thyroid cancer [80], it has not led to corresponding preclinical trials. Likewise, despite strong evidence for STAT3’s involvement in anaplastic thyroid cancer [47], related trials such as NCT03195699 have not specifically prioritized EMT/MET in other forms of poorly differentiated thyroid cancer.

Preventing lineage plasticity through epigenetic modulation (e.g., EZH2, LSD1, or IDH1/2 inhibitors) can restrict therapy-induced transdifferentiation. Differentiation therapy aims to lock tumor or cancer stem cells into less aggressive states, as demonstrated by all-trans retinoic acid in leukemia and emerging IDH/LSD1 inhibitors in solid tumors [81,82].

The STING (Stimulator of Interferon Genes) pathway offers another innovative strategy by linking innate and adaptive immunity through type I interferon production. The STING pathway is activated by agonists such as cyclic dinucleotides (CDNs) like ADU-S100 and synthetic agonists like MK-1454, which mimic the natural ligand cGAMP. While dedicated clinical data remain limited, early-phase trials such as NCT04592484 evaluating CDK-002 (exoSTING) in ATC and advanced solid tumors highlight growing interest in this approach. Preliminary findings from trials of MK-1454 and ADU-S100 in other malignancies, including partial responses in undifferentiated thyroid cancer, further support its relevance (NCT03010176, NCT02675439). Therefore, STING could be considered as an emerging strategy because its agonists (e.g., ADU-S100) are uniquely positioned not just as immune activators, but also as crucial initiators of the entire anti-tumor immune cascade, connecting the initial innate response to the more specific and durable adaptive T-cell response. Early-phase clinical trials (Phase I/II) with STING agonists have shown promising results, demonstrating their ability to enhance T-cell infiltration within various solid tumors. STING activation synergizes powerfully with ICIs and TKIs, amplifying anti-tumor immune responses and improving therapeutic outcomes [83]. Innovative approaches include delivering STING agonists via pH-sensitive nanoparticles designed to release their payload specifically in the acidic tumor microenvironment, thereby reducing systemic off-target effects. Furthermore, engineering dendritic cells with enhanced STING activity for use in personalized vaccines can significantly boost T-cell priming and anti-tumor immunity. The primary challenge for STING agonists is that systemic activation can cause inflammatory toxicity. The proposed solutions, such as pH-sensitive targeted nanoparticles and AI-designed agonists, directly address this, reinforcing that potent immune activators require advanced delivery and design technologies to confine their activity to the tumor microenvironment.

Advancements in ICI engineering, including bispecific antibodies that co-target PD-L1 and tumor-specific antigens such as thyroid-stimulating hormone receptor (TSHR) or calcitonin, could enhance both immune activation and tumor specificity. Engineering Chimeric Antigen Receptor (CAR) T cells or Natural Killer (NK) cells to not only disrupt PD-1/PD-L1 interactions but also target thyroid-specific antigens (e.g., TSHR, TPO, GFRa4, etc.) offers a highly personalized and potent cellular therapy. Utilizing mRNA vaccines that encode anti-PD-L1 antibodies or PD-1 inhibitors, delivered via lipid nanoparticles, can induce localized and potent immune activation within the TIME as well (summarized in Table 2).

Aberrant WNT/β-catenin signaling is another major driver of immune exclusion in thyroid tumors, especially ATC. Unlike PD-1/PD-L1 which inactivates T cells, or TGF-β which suppresses them, WNT/β-catenin creates a distinct, physical barrier mechanism. Recent evidence [84] shows that canonical Wnt/β-catenin signaling is almost universally activated in ATC, primarily via ligand overexpression rather than mutation. Functional inhibition of this pathway using the small molecule pyrvinium pamoate significantly reduced ATC cell viability in organoid cultures and xenograft models, providing a strong preclinical rationale for targeting Wnt signaling in thyroid cancer. Novel therapeutic strategies include PROTACs (proteolysis-targeting chimeras) that degrade β-catenin, CRISPR-based epigenetic silencing of WNT genes (e.g., CTNNB1), and tumor-targeted nanoparticle delivery of WNT inhibitors such as WNT-974. Notably, β-catenin–targeting PROTAC peptide has shown durable suppression of Wnt-dependent tumor growth in intestinal cancer models [85]. In parallel, genome-wide CRISPR-Cas9 screening identified CTNNB1 as a critical vulnerability in Wnt-driven tumors [86], with its silencing impairing tumor viability and Wnt transcriptional output. Tumor-targeted nanoparticles (e.g., pH-sensitive, ligand-coated) are instrumental in delivering various therapeutic agents, including TLR/STING agonists, TGF-β inhibitors, or WNT inhibitors. Their primary advantage lies in minimizing systemic toxicity while significantly enhancing specificity and concentration of the therapeutic payload within the TIME. Leveraging β-catenin-targeting PROTACs and CTNNB1 silencing, potentially via nanoparticle-based delivery systems, could disrupt the immuno-resistant architecture of the ATC microenvironment and sensitize tumors to immunotherapies. Future studies should prioritize spatial multi-omics in annotated human cohorts (>100/subtype) to resolve microenvironmental heterogeneity and design biomarker-stratified trials to dissect TIME-modulatory effects. The success of these next-generation interventions relies heavily on cutting-edge enabling technologies including AI and machine learning to optimize drug design, patient stratification, and combination therapy planning.

While a compelling association exists between gut dysbiosis and thyroid cancer, current evidence remains largely correlative and mechanistically superficial. Significant investment in establishing rigorously controlled cohorts including larger, multi-center studies with standardized methodologies, detailed phenotyping (TC subtype, stage, genetics, diet, comorbidities), and appropriate control groups (HC, benign disease, other cancers) are needed. Intervention trials targeted at determining if modulating the microbiome (probiotics, prebiotics, FMT, antibiotics) beyond symptom management influence thyroid cancer incidence, progression, or treatment response are the need of hour (all summarized in Table 2).

### Limitations of This Review

While this manuscript presents a comprehensive genetic driver–metabolic–immune framework for potential thyroid cancer therapeutics, it does not account for the emerging role of the neural–immune–tumor triad within the tumor microenvironment. Neural components such as tumor-associated nerves and neurotransmitters can significantly influence tumor progression by promoting angiogenesis (e.g., via catecholamines), enabling metastasis through perineural invasion, and modulating immune responses through axon guidance molecules that suppress T cell activity. Furthermore, the manuscript does not explore the epigenetic mechanisms underlying iodine refractoriness, a critical barrier to effective radioiodine therapy. The potential of epigenetic modulators such as histone deacetylase inhibitors and DNA demethylating agents, to restore iodine uptake in dedifferentiated thyroid cancers represents a therapeutic avenue for future investigation.

## 10. Conclusions

This review examines recent literature on the etiopathology of thyroid cancer, focusing on genetic abnormalities, tumor microenvironment interactions, and their translational impact on diagnostics and therapeutics. While our understanding of the genetic landscape of thyroid cancer is robust and drives targeted diagnostic and therapeutic development, insights into the thyroid tumor microenvironment and its role in diagnosing and/or modulating cancer behavior remain comparatively limited.

## Figures and Tables

**Table 1:** A simplified conceptual pathway-centric overview of tumor subtypes.

Pathway	Primary Subtype	Key Drivers	Clinical Behavior	TMB	Immune Infiltration	PD-L1 Expression
MAPK	Papillary (PTC)	BRAF, RET/PTC, RAS	Well-differentiated	Low (<1 mut/Mb)	Variable (higher in BRAF like)	Moderate
PI3K/AKT	Follicular (FTC)	RAS, PIK3CA, PTEN loss	Well-differentiated	Low (<1 mut/Mb)	Low	Low (inconsistent)
MAPK + PI3K/AKT	PDTC/ATC	Multiple mutations	Aggressive	Intermediate	Heterogeneous	Variable
RET	Medullary (MTC)	RET mutations	Neuroendocrine	Low	Variable (metastatic-high)	Low
Multiple	Anaplastic (ATC)	BRAF/RAS + TP53, TERT, PIK3CA co-mutations	Highly aggressive	Highest among thyroid cancers	High (M2 macrophages)	High

**Table 2:** Summary of therapeutic strategies in thyroid cancer: phase of investigation, subtype applicability, limitations, and outstanding questions.

Therapeutic Strategy	Agents	Target/Pathway	Phase	Thyroid Subtype Applicability	Supporting Evidence	Limitations	Outstanding Questions
**KINASE INHIBITORS**							
BRAF + MEK Inhibition	Dabrafenib + Trametinib	BRAF/MEK/MAPK	Phase II (PTC)/FDA-approved (ATC)	BRAFV600E-mutant ATC; BRAFV600E PTC	FDA-approved for BRAF-mutant ATC; NCT04554680 confirmed therapeutic benefit (excludes aggressive histotypes); PFS > 15 mo in PTC; OS ~14 mo in ATC despite 56% ORR.	Phase III excludes aggressive histotypes. Resistance via MAPK reactivation (KRAS/NRAS mutations, BRAF splice variants), PI3K/AKT activation (IGF-1R upregulation, PTEN loss), hypoxia-induced HIF-1α, and immune evasion: all preclinically documented but incompletely mapped in patients.	Do combinatorial BRAFi + MEKi ± PI3Ki/ICI strategies durably overcome the multiple documented resistance mechanisms? Which molecular biomarkers predict sustained response vs. early resistance?
Neoadjuvant Triple Therapy (DTP)	Pembrolizumab + Dabrafenib + Trametinib	PD-1 + BRAF + MEK	Phase II (single arm; no control)	BRAFV600E-mutant ATC only	74% surgical conversion vs. ~5% historical; added ICI to BRAFi/MEKi backbone enabled majority to undergo resection.	Single-arm design cannot isolate pembrolizumab’s marginal contribution over dabrafenib/trametinib alone. Historical controls not BRAF-mutation stratified (biological non-equivalence). Small sample size. Absence of long-term survival data.	What is pembrolizumab’s independent contribution to surgical conversion? Does resection convert to a survival benefit? These questions require a randomized design.
Selective RET Inhibition	Selpercatinib, Pralsetinib	RET kinase (MTC/RET-fusion cancers)	Phase I/II (regulatory approval: MTC and RET-fusion cancers)	RET-mutant MTC; RET-fusion PTC/PDTC	ORR ~80% with selective RET inhibitors in MTC; major advance from nonselective multikinase inhibitors (vandetanib, cabozantinib).	Solvent-front resistance mutations (G810, most common) develop predictably. Src-mediated bypass documented in preclinical models only; no completed randomized thyroid-specific combinatorial data.	Does RET + Src co-inhibition overcome acquired resistance clinically? Are resistance mechanisms consistent across RET-mutant subtypes, or is subtype-stratified trial design needed?
PI3K/AKT/mTOR Inhibition	GDC-0941 (PI3Ki); MK-2206 (AKTi)	PI3K/AKT/mTOR	Preclinical/Early Phase I (non-thyroid-specific)	ATC, PDTC (PIK3CA-mutant; PTEN-loss)	PIK3CA mutations and PTEN loss prevalent in ATC/PDTC; pathway drives VEGF, angiogenesis, metabolic reprogramming; MAPK/PI3K crosstalk underpins resistance to single-pathway inhibition.	No completed randomized thyroid-specific data. MAPK/PI3K crosstalk means single-pathway inhibition triggers compensatory signaling; vertical inhibition strategies untested in thyroid cancer.	Can vertical inhibition (BRAF + MEK + PI3K/AKT) overcome crosstalk-driven resistance without unacceptable toxicity in thyroid cancer patients?
Src Kinase Inhibition	Saracatinib (AZD0530); PP2; TPX-0046; Src-3 Inhibitor-2 (SI-2)	Src/SFK (convergence resistance hub)	Preclinical (PTC, ATC, MTC)/Vandetanib FDA-approved MTC (multikinase -not Src-selective)	ATC, MTC (Src-overexpressing); BRAFi-resistant PTC	Src overexpression in ATC/MTC; RET-Y981 phosphorylation activates Src in MTC; Src-3/NCOA3 amplified in ATC.	Activating Src mutations rare. Src functions as convergence node, not primary oncogenic driver. Spatial heterogeneity (core invasion vs. peritumoral M1 survival benefit) complicates systemic inhibition. No thyroid-specific Src monotherapy trial completed.	At what disease stage and co-mutation context does Src inhibition provide benefit vs. risk? Combination with BRAFi/PI3Ki untested clinically in thyroid cancer.
JAK/STAT3 Inhibition	Ruxolitinib, Tofacitinib (JAKi); TTI-101 (STAT3i)	JAK1/2—STAT3 axis	Preclinical (thyroid-specific)/Phase I/II (other cancers; BRAFi-resistant context)	ATC, PDTC (JAK1-mutant, IL-6-high); BRAFi-resistant BRAFV600E cancers	JAK-STAT activation as BRAFi resistance mechanism; STAT3 oncogenic in ATC via IL-6/PD-L1/Treg expansion; STAT3 tumor-suppressive via p21-mediated senescence in early PTC.	STAT3 is tumor-suppressive in early p53-wild-type PTC but oncogenic in p53-mutant ATC. Universal inhibition risks harm in early-stage disease. TP53 mutation status rarely reported as stratifying variable in published thyroid STAT3 studies creating apparently paradoxical pooled evidence.	Can STAT3 inhibition be safely restricted to p53-mutant/ATC contexts? A biomarker-stratified trial by TP53 status and disease stage is a prerequisite for safe clinical translation.
**TARGETED PATHWAY INHIBITORS**							
Wnt/β-catenin Pathway Inhibition	WNT974 (Porcupine inhibitor); Vantictumab; Pyrvinium pamoate; HDAC inhibitors	Wnt/β-catenin (ligand-driven activation in ATC)	Preclinical (thyroid-specific: organoid + xenograft in ATC)/Phase I (other cancers: WNT974)	ATC only (ligand-driven activation); Not broad thyroid cancer	Canonical Wnt activated in ATC via ligand overexpression -not CTNNB1 mutation; pyrvinium pamoate reduced ATC viability in organoids and xenografts. This distinction means CTNNB1-targeted strategies inappropriate; ligand-trap/Porcupine inhibition mechanistically rational for ATC.	Wnt5a paradox: tumor-suppressive in FTC, pro-metastatic in PTC; Systemic Wnt inhibition: gut epithelial and bone toxicity. No thyroid-specific clinical trial has been initiated.	Does ligand-trap or Porcupine inhibition translate to clinical ATC benefit? This represents the largest translational gap, a thyroid-specific trial is the immediate priority.
TGF-β Pathway Inhibition	SB431542 (preclinical); systemic TGFβRII inhibitors (other cancers)	TGF-β/SMAD signalling	Preclinical (thyroid-specific)/Phase I/II (non-thyroid)	ATC (SMAD2-S100A4-MMP2/9 axis); BRAFV600E PTC (NIS repression reversal)	TGF-β1 drives ATC invasion via SMAD2-S100A4-MMP-2/9; BRAFV600E-induced TGF-β secretion represses NIS → RAI resistance; M2 macrophage polarisation and SNAIL/SLUG upregulation.	TGF-β is tumor-suppressive in early thyroid cancer. Systemic inhibition risks releasing early tumor cells from growth restraint and triggering autoimmunity/inflammatory toxicity. No thyroid-specific clinical data.	Can local/nanoparticle-targeted TGF-β inhibition avoid systemic tumor-suppressor disruption? Can TGF-β inhibition restore RAI sensitivity in BRAFV600E PTC clinically. A high-priority unmet need with clear biological rationale?
**IMMUNE CHECKPOINT INHIBITORS**							
Anti-PD-1/PD-L1 Monotherapy	Pembrolizumab, Nivolumab, Atezolizumab	PD-1/PD-L1 axis	Phase II (deepest clinical evidence in thyroid; no thyroid-specific randomised trial)	ATC (primary); BRAFV600E PTC (combinatorial context); RAI-refractory DTC	PD-L1 expression highest in ATC; BRAFV600E upregulates PD-L1 via MAPK-dependent transcription; RAS-mutant tumors: lower PD-L1 → ‘cold’ TME → likely resistant to monotherapy.	PD-L1 expression heterogeneous and not validated as predictive biomarker in thyroid-specific trials. Majority of data with small thyroid cancer cohorts. No thyroid-specific randomised controlled trial.	Which thyroid molecular subtypes benefit from ICI monotherapy vs. combination? Is PD-L1 IHC a reliable predictive biomarker in thyroid cancer, or does spatial/temporal heterogeneity render it insufficient?
Bispecific Antibodies (PD-L1 × TSHR/Calcitonin)	Anti-PD-L1 × anti-TSHR or anti-calcitonin bispecifics	PD-L1 + thyroid-specific antigens (TSHR, calcitonin)	Conceptual/Preclinical (thyroid-specific context only)	PTC/DTC (TSHR-expressing); MTC (calcitonin-expressing)	Rationale: thyroid antigen specificity of TSHR and calcitonin to improve tumour selectivity. Bispecific technology established in haematologic malignancies. No thyroid-specific bispecific data.	No clinical data in thyroid cancer. Thyroid antigen expression may be lost in dedifferentiated/ATC tumors. Off-target thyroid tissue targeting risks autoimmune hypothyroidism.	Do thyroid-specific bispecifics improve tumour selectivity over PD-L1 monotherapy in a clinically meaningful way? Requires thyroid-specific preclinical proof-of-concept before first-in-human studies.
**CELLULAR THERAPIES**							
CAR-T/CAR-NK Cell Therapy	Anti-TSHR CAR-T cells; Anti-GFRα4 CAR-NK cells	TSHR, TPO, GFRα4	Preclinical/Conceptual (thyroid-specific—no clinical trial data)	DTC (TSHR-expressing); MTC (GFRα4-expressing)	Rationale from thyroid antigen expression profiles. No completed thyroid-specific CAR-T trial.	Solid tumour immunosuppressive TME limits CAR-T infiltration and persistence. Antigen loss/downregulation common in advanced thyroid cancer. Cytokine release syndrome risk.	Can CAR-T/CAR-NK cells penetrate and survive in the immunosuppressive thyroid tumour TME? Patient-derived organoid/xenograft validation required before first-in-human thyroid studies.
mRNA Vaccine (anti-PD-L1/PD-1 encoding)	LNP-encapsulated mRNA encoding anti-PD-L1 antibodies or PD-1 inhibitors	PD-1/PD-L1 (intratumoral local expression)	Conceptual/Early Preclinical (non-thyroid-specific)	Theoretically broad; no subtype specificity established	Platform rationale from mRNA vaccine technology (COVID-19; emerging oncology trials in other cancers). No thyroid-specific data.	No thyroid-specific data. Delivery to thyroid tumour TME unproven. Immunogenicity of repeated dosing unclear. Entirely conceptual in thyroid cancer context.	Can intratumoral mRNA vaccine delivery achieve sustained local ICI expression in thyroid tumours?
**INNATE IMMUNITY MODULATORS**							
TLR Agonists (TAM Reprogramming: M2→M1)	Entolimod (TLR5/flagellin derivative); MPLA (TLR4 agonist)	TLR5/TLR4 → M2→M1 macrophage reprogramming	Preclinical (immunocompetent murine models only)/Phase I/II (non-thyroid cancers)	ATC (cold TME; high M2-TAM burden-primary rationale)	TLR5 agonist synergy with ICIs demonstrated in immunocompetent murine tumour models. MPLA safer TLR4 profile vs. LPS. Rationale: heating immunologically cold ATC TME.	ICI synergy tested only in immunocompetent mice; does not model human T-cell exhaustion in advanced ATC, a biologically distinct state. Systemic TLR activation risks cytokine storm/sepsis-like syndrome. No thyroid-specific human data.	Does TLR agonist-mediated M1 reprogramming overcome T-cell exhaustion in advanced human ATC, which differs fundamentally from immunocompetent murine models? Human thyroid-specific data entirely absent.
STING Pathway Activation	ADU-S100; MK-1454; CDK-002 (exoSTING); pH-sensitive nanoparticle delivery systems	STING/cGAS → Type I IFN → T-cell priming	Phase I/II (early; partial responses in undifferentiated TC: NCT03010176, NCT02675439); NCT04592484 ongoing in ATC	ATC (primary; NCT04592484); potentially applicable across subtypes	Phase I/II: T-cell infiltration enhancement across solid tumours; partial responses in undifferentiated thyroid cancer in basket trials (NCT03010176, NCT02675439); NCT04592484 evaluating CDK-002 in ATC (ongoing; results awaited).	Systemic STING activation causes inflammatory toxicity. Thyroid-specific efficacy data limited to basket trial subgroups/case reports. pH-sensitive nanoparticle delivery remains preclinical concept. Most ATC-specific data hypothesis-generating only.	Does intratumoral/nanoparticle-targeted STING activation avoid systemic toxicity while retaining efficacy in ATC? Results of NCT04592484 are awaited; thyroid-specific randomised data remain absent.
**METABOLIC THERAPIES**							
Metabolic-Immune Circuit Breakers (IDO1/GLS1 + ICI)	Epacadostat (IDO1i) + Pembrolizumab (ECHO-202); CB-839 (GLS1i); 2-DG (glycolysis inhibitor)	IDO1/tryptophan catabolism; GLS1/glutaminolysis; aerobic glycolysis	Phase I/II (ECHO-202: non-thyroid-specific)/Preclinical (thyroid-specific concept)	BRAFV600E PTC and ATC (IDO1-overexpressing); broadly applicable metabolic concept	ECHO-202: 55% ORR with epacadostat + pembrolizumab in immunotherapy-naïve solid tumour patients; IDO1 overexpression associated with Treg infiltration in BRAF-mutant PTC/ATC (small IHC studies only).	Epacadostat failed in KEYNOTE-252 (Phase III, melanoma) thereby raises the fundamental question whether IDO1 inhibition independently contributes to ICI response, or whether ECHO-202’s ORR reflected pembrolizumab activity alone. IDO1 overexpression in thyroid: small single-institution IHC studies without validated scoring methodology. No thyroid-specific metabolic ICI trial initiated.	Does IDO1 inhibition contribute independently to ICI response in thyroid cancer, given the KEYNOTE-252 phase III failure signal in a more ICI-responsive tumour type? Thyroid-specific phase II with IDO1-stratified enrollment is required before ‘Metabolic-Immune Circuit Breaker’ moves from hypothesis to strategy.

## Data Availability

No new data were created or analyzed in this study. Data sharing is not applicable to this article.
